# New Opportunities in Glycan Engineering for Therapeutic Proteins

**DOI:** 10.3390/antib11010005

**Published:** 2022-01-10

**Authors:** Xiaotian Zhong, Aaron M. D’Antona, John J. Scarcelli, Jason C. Rouse

**Affiliations:** 1 BioMedicine Design, Medicinal Sciences, Pfizer Worldwide R&D, 610 Main Street, Cambridge, MA 02139, USA; aaron.dantona@pfizer.com; 2 BioProcess R&D, Biotherapeutics Pharmaceutical Sciences, Medicinal Sciences, Pfizer Worldwide R&D, 1 Burtt Road, Andover, MA 01810, USA; john.scarcelli@pfizer.com; 3 Analytical R&D, Biotherapeutics Pharmaceutical Sciences, Medicinal Sciences, Pfizer Worldwide R&D, 1 Burtt Road, Andover, MA 01810, USA; jason.rouse@pfizer.com

**Keywords:** glycosylation pathways, N-acetyl-galactosamine, mannose-6-phosphate, lysosomal degradation, Fab glycans, antibody diversification, sialylation, glycome, O-linked glycans, therapeutic proteins

## Abstract

Glycans as sugar polymers are important metabolic, structural, and physiological regulators for cellular and biological functions. They are often classified as critical quality attributes to antibodies and recombinant fusion proteins, given their impacts on the efficacy and safety of biologics drugs. Recent reports on the conjugates of N-acetyl-galactosamine and mannose-6-phosphate for lysosomal degradation, Fab glycans for antibody diversification, as well as sialylation therapeutic modulations and O-linked applications, have been fueling the continued interest in glycoengineering. The current advancements of the human glycome and the development of a comprehensive network in glycosylation pathways have presented new opportunities in designing next-generation therapeutic proteins.

## 1. Introduction

Glycan modification [1,2,3,4], in mammalian glycoproteins, glycolipids and recently in RNAs [5], represents the most complex and diverse networks and pathways for post-translational modifications. The tremendous structural diversity of glycan polymers are synthesized without a template, but rather through a sequential step addition by compartmentally restricted cellular glycosylation machineries which employ around 700 genes encoding glycotransferase enzymes, transporters and chaperones required for establishing the ensemble of glycans [1,6]. Apart from the non-enzymatic glycation between glucose and lysine/arginine [7] as well as cytosolic and nuclear O-GlcNAcylation [8], protein glycosylation processes involve sequentially orchestrated modification reactions in the metabolic networks of the endoplasmic reticulum (ER) and the Golgi during protein trafficking. It has been estimated that the known glycome and the glycosylation network are generated through 16 distinct glycosylation pathways according to sugar–protein linkages, initial monosaccharides linked to proteins, and unique initiating enzymes [1].

Glycan attachments to protein are generally classified into four major types. N-linked glycosylation is through asparagine (Asn) that is initiated at the ER by the *en bloc* transfer of core glycans via oligosaccharyltransferase (OST) and further modified by various glycoenzymes and glycotransferases in the ER and the Golgi [1,2,3,9]. O-linked glycosylation involves covalent modification to the hydroxyl groups of serine (Ser), threonine (Thr), or tyrosine (Tyr) with direct attachments of seven different sugars including N-acetyl-galactosamine (GalNAc), L-fucose (Fuc), N-acetyl-D-glucosamine (GlcNAc), D-mannose (Man), D-glucose (Glc), D-xylose (Xyl), and D-galactose (Gal). GalNAc-type and Xyl-type O-linked glycosylation start at the Golgi by polypeptide GalNAc transferases (GALNTs) and O-xyltransferases (XYLTs), respectively. Fuc, Glc, and GlcNAc types of O-linked glycosylation are initiated in the ER. Mammalian Man-type O-linked glycosylation is initiated in the ER and further modified in the Golgi. The other two ways for glycan attachments are glypiation through GPI linkage and C-linked to tryptophan (Trp) [1].

The natural building blocks for glycans in mammals are 10 monosaccharides including D-glucuronic acid (GlcA), D-ribose (Rib), Fuc, Glc, GlcNAc, Gal, GalNAc, Man, N-acetylneuraminic acid (Neu5Ac), and Xyl, which can be derived from the corresponding dolichol-linked donors or activated donor sugar nucleotides [1,2,3]. The structural diversification of glycans through the sequential addition of monosaccharides mostly occur in the Golgi for oligosaccharide extending, branching, and capping. The final glycan structures are determined by glycosyltransferases’ kinetic properties, their compartmental distributions along the sequential biosynthetic routes, as well as factors such as substrate availability and actions of protein chaperones and glycosidases.

**Figure 1:**
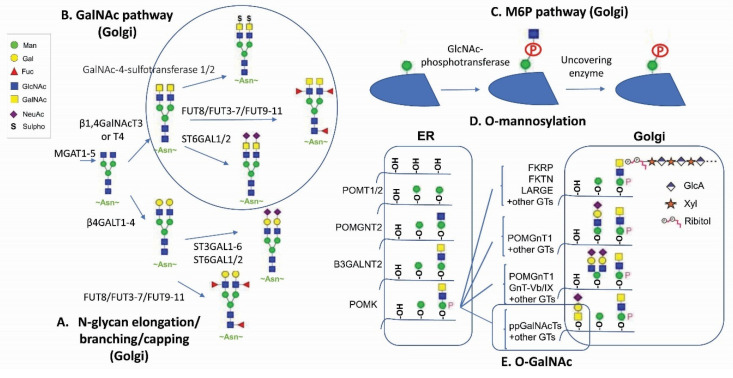
Major human glycan pathways. (**A**) N-glycan elongation, branching, and capping pathways. (**B**) GalNAc pathway [14]. (**C**) M6P pathway [15]. (**D**) O-mannosylation [16]. (**E**) O-GalNAc pathway (circled).

**Figure 2:**
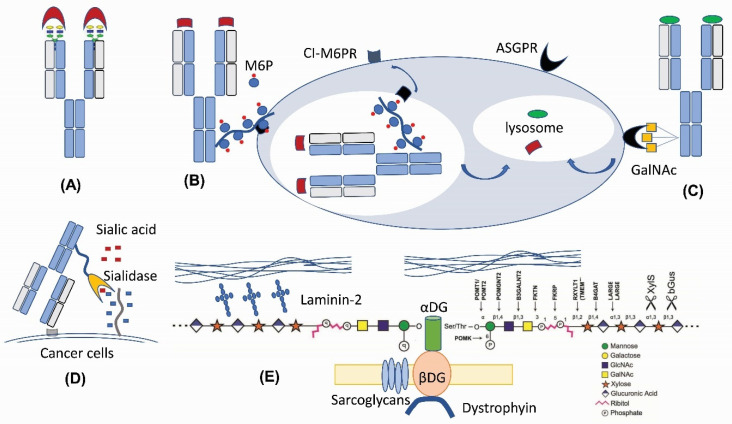
New therapeutic applications of N-linked and O-linked glycan modifications. (**A**) Fab N-glycan for the antibody diversity [17]. (**B**) M6P-mediated lysosomal degradation [18]. (**C**) GalNAc-mediated lysosomal degradation [19,20,21]. (**D**) Antibody–sialidase fusions or conjugates [22,23]. (**E**) O-mannosylation matriglycan as a functional decoration for α-Dystroglycan [24,25].

Therapeutic proteins, such as antibodies and recombinant fusion proteins, are glycoproteins in which glycan modifications are often considered critical quality attributes and can be engineered for therapeutic efficacy and safety improvements (according to several reviews [6,10,11,12,13]). With a global view on the human glycome being established and a deeper understanding on glycosylation pathways, new opportunities in harnessing human protein glycosylation functions are emerging (Figure 1). This article highlights new applications of GalNAc and mannose-6-phosphate (M6P) glycan modification in protein therapeutics (Figure 2). New findings on antibody repertoire glycan diversification, O-linked mannosylation, glycan remodeling on branching, sialylation, and fucosylation were also discussed.

## 2. Glycans as an Unconventional Strategy for Antibody Diversification

N-linked glycans are present in 15–25% of human IgG antibodies’ variable domain (heavy chain variable domain (VH) or light chain variable domain (VL)) regions [26,27]. These N-glycosylation sites encoded by the V-region genes (so-called Fab N-glycans) are a result of somatic hypermutation [26,28,29], because very few germline alleles carry N-glycosylation consensus sequences (NXS/T) [30]. In recent years, more and more evidences indicate that Fab N-glycans can influence antibody binding affinity. Several mechanisms on how N-glycan in antibody V-regions impacts epitope binding have been proposed, including the bulk size of N-glycan to fill out the space between the antigen epitope and the antibody paratope [31], charge–charge interaction between N-glycan sialic acids and the antigen [17,28], and through steric hinderance effects that affect the binding [32]. The IgG4 subclass has the highest prevalence of V-region glycosylation (44% versus 11%–15% in other subclasses) [28]. IgE has a two-fold higher propensity for Fab glycans than IgA or IgG1, suggesting that elevated Fab glycosylation might be a hallmark of Th2-like responses [33]. A large portion of autoantibodies in rheumatoid arthritis and certain B-cell lymphomas were found to contain Fab N-glycans [34,35,36], which are also present in human anti-idiotype autoantibodies to adalimumab and infliximab [28]. Removing N-glycans from the complementarity-determining regions (CDRs) of antibodies can lead to a significant decrease in the antibody binding affinity [28,37,38]. Removing N-glycan located within the antigen-binding sites of a human IgG alloantibody decreases its neutralization towards factor VIII (FVIII) procoagulant activity without losing its binding affinity, suggesting that its Fab glycan blocks the interaction between FVIII and the chaperone partner through steric hinderance [32]. Fab glycans in the framework or constant regions play additional roles in increasing antibody stability [29] and in vivo half-life [39].

The structure of N-glycans within the V region are different from those rigid under-sialylated biantennary Fc-glycans attached to Asn297 in the Fc region, because they are typically surface-exposed α2,6-linked sialylated complex biantennary glycans [37,40,41]. The negatively charged sialic acid on these V-region glycans have been found to contribute to the increased binding affinity [28,38,40]. This data indicate that the introduction of N-linked glycans to variable domains is an additional layer for immune repertoire diversification [17].

Engineering N-glycans into antibody-binding sites has been utilized for therapeutic rational design (Figure 2A). Engineering an N-linked consensus site into an ibalizumab light chain recognizes human immunodeficiency virus (HIV)’s envelope glycoprotein gp120 with a loss of an N-glycan in the V5 loop, which is otherwise resistant to the HIV-1-neutralizing activity [31]. Similarly, introducing Fab glycans into adalimumab enhances the TNFα binding of two antibody glycovariants by two-fold [28]. Introducing Fab N-glycans can be a way to decrease antigen-binding poly-reactivity and self-reactivity [42,43]. The introduction of an N-linked glycan into an antibody-variable domain also has been employed for improving antibody solubility [44,45]. Although engineering in Fab N-glycosylation can increase manufacturing challenges, the high degree of conformational dynamics from glycans can enhance the chemical diversity of antibody paratopes and thus the functionalities.

## 3. GalNAc Binder—A New Application Based on Previous Findings

Therapeutic antibodies can exert biological actions on signal transduction pathways by blocking interactions between receptors and ligands. Three recent studies reported a new therapeutic mechanism by conjugating tri-GalNAc to antibodies for directing the lysosomal degradation of several therapeutic ligands and receptors [19,20,21]. These so-called lysosome-targeting chimeras (LYTACs) are capable of inducing a rapid internalization and degradation of membrane targets and soluble targets based on the binding to the liver-specific asialoglycoprotein receptor (ASGPR) and lysosome machineries (Figure 2C).

Naturally, GalNAc residues (Figure 1B,E) can be added to proteins through either N-glycan LacdiNAc modification or O-linked GalNAc addition [1,3,6]. The tight binding to ASGPR or mannose receptor (MR) has been reported for N-glycan LacdiNAc modified proteins [46]. LacdiNAc is a less common N-linked glycan structure [14,47,48,49,50]. It contains the unique GalNAcβ1-4GlcNAcβ unit, which can be additionally sulfated, fucosylated and sialylated. About 12 glycoproteins are confirmed with N-linked LacdiNAc glycans, i.e., luteinizing hormone. β1,4GalNAcT3 [51] and β1,4GalNAcT4 [52] account for GalNAc transfer and have a broad tissue expression coverage including fetal kidney and brain. It has been reported that adding a carboxyl-terminal 19-amino-acid α-helix stretch with several basic amino acids is sufficient to mediate GalNAc transfer to N-linked oligosaccharides [47,53,54]. Other GalNAc motifs involve three structural loops with aromatic side chains [50], as well as additional unidentified motifs [54].

Since LYTAC molecules conjugated with GalNAc can be targeted for lysosomal degradation, fusing the GalNAc transfer motif to the termini of antibodies or therapeutic fusion proteins should enable the LacdiNAc modification on these proteins during mammalian cell culturing. HEK293 cells express the key glycoenzymes of β1,4GalNAcT3, β1,4GalNAcT4, and GalNAc-4-sulfotransferases-1 and -2, and a stable production in HEK293 has generated clinical and commercial biotherapeutics [55]. In fact, because CHO cells either lack or do not express several glycosyltransferases, therapeutic proteins such as recombinant human erythropoietin are found to be LacdiNAc-modified when expressed in HEK293 cells, but not in CHO cells [55,56]. Alternatively, CHO cells with necessary glycoenzymes can be engineered for LacdiNAc modification [47].

## 4. M6P—A Lysosomal Route for Non-Lysosomal Enzymes

M6P modification (Figure 1C) in specific N-linked glycans serves as a recognition signal for lysosomal routing [11,15,57]. When lysosomal hydrolases synthesized in the ER are transported to the *cis*-Golgi network, they are selectively modified by a two-step reaction. GlcNAc-1-phosphotransferase transfers a GlcNAc-1-phosphate residue from UDP-GlcNAc to C6-positions of specific mannoses in high-mannose N-glycans of lysosomal hydrolases. The GlcNAc-1-phosphotransferase is a Golgi hexameric transmembrane enzyme encoded by two different genes, i.e., *GNPTAB* and *GNPTG* [15]. Defects in this key enzyme causes lysosomal storage disease mucolipidosis II and III. The second step of M6P generation is catalyzed by an N-acetylglucosamine-1-phosphodiester α-N-acetyl-glucosaminidase (also known as “uncovering enzyme”) for the removal of the terminal GlcNAc to expose the signal. The uncovering enzyme is a tetrameric type I membrane protein cycling between the *trans*-Golgi network and the plasma membrane. No pathological conditions are associated with the loss of its enzymatic activity.

In the *trans*-Golgi network, the M6P modification allows for the segregation of lysosomal hydrolases from other trafficking proteins through a selective binding to two M6P receptors, i.e., the cation-independent M6P receptor (CI-MPR) and/or the cation-dependent M6P receptor (CD-MPR) [15,57]. The clathrin-coated ligand receptor complex transport vesicles bud off and fuse with late endosomes. At the low pH of the late endosome (Figure 2B), the M6P receptors dissociate from the ligands and be recycled back to the *trans* Golgi network.

The therapeutic application of M6P modification is the lysosomal delivery of the enzyme replacement therapy for lysosomal diseases, such as Fabry disease, mucopolysacharidosis I, II, and VI, and Pompe disease [58]. High-affinity M6P analogues with good stability, such as mannose-6-phosphonate (M6Pn), could be synthesized [59] and conjugated, like the M6P-containing oligosaccharides [60], to recombinant enzymes for decreasing the effective dose for less accessible tissues. M6P is also present in glycoprotein D of herpes simplex virus (HSV) for virus entry into cells [61]. Recombinant CI-M6PR and pentamannose-phosphate are used to block HSV plaque formation [62]. Most recently, M6P has been exploited for lysosomal degradation [18]. Because the 6-phosphoester of M6P can undergo hydrolysis in human serum, the phosphatase-inert serine-O-M6Pn glycopeptide is conjugated to an antibody to form a different kind of LYTACs that interacts with CI-M6PR for shuttling to the lysosomal compartment for the degradation of extracellular proteins engaged by the antibody component of the conjugates. For the biological production of M6P-modified glycoproteins, one strategy is to utilize engineered yeast cells to synthesize Man-P-6-Man glycans, in which phosphate-capped Man residues can be subsequently removed by a newly discovered α-mannosidase to generate M6P-modified human lysosomal enzymes [63]. These new tools and new findings should enable further glycoengineering of next-generation biologics for lysosomal targeting.

## 5. O-Linked Glycan: New Tricks for an Old Player for Biological Systems

GalNAc-type-O-glycosylation of Ser/Thr is the most common type of O-linked glycosylation, which can be initiated by up to 20 different GALNTs, with a portion seemingly having protein-specific functions. For example, some of these enzymes are responsible for generating simple truncated O-linked glycans known as cancer-associated Tn antigens [1,6]. GALNT3 uniquely modulates the processing site of FGF23 that regulates phosphate homeostasis [64]. GALNT11 specifically modifies the low-density lipoprotein receptor-related receptor family and enhances ligand binding [65]. GalNAc-type-O-glycosylation in recombinant TNFR:Fc fusion protein has a significant impact on its pharmacokinetics [66]. O-glycosylation affects ADAM proteases [67], β1-adrenergic receptor activation [68], and atrial natriuretic peptide potency [69]. O-glycans attached to neuropeptide Y and the glucagon family members modulate receptor activation properties and extend half-lives, demonstrating the importance of O-glycosylation in peptide hormones. O-Fucosylation and O-glucosylation stabilized the folding of EGF-like and thrombospondin type 1 repeat domains [70,71]. Recently, a proteomic-based strategy uncovers that one-third of 279 classified peptide hormones carry O-glycans and that many of these identified O-glycosites are predicted to serve roles in proprotein processing, receptor interaction, biodistribution, and biostability [72]. Since O-glycans can impact biotherapeutics in a number of ways, such as impacting pharmacokinetics [66], decreasing the binding affinity of peptide–antibody fusions [73], and unexpected O-glycosylation in antibody fusion linkers for manufacturing issues, understanding this old player for the biological systems could help to develop new tricks for biotherapeutics applications.

Man-type O-linked glycosylation takes place in both ER and Golgi (Figure 1D). The human protein-O-mannosyl transferase POMT1/2, the initiating heteromeric complex, specifically recognizes the central mucin-like domain within α-dystroglycan (α-DG) [16] and a very limited number of other substrates [74]. In fact, this rare type of O-linked mannosylation found in α-DG is called matriglycan. It contains phosphorylation, ribitol, GlcA, and Xyl repeats that interact with extracellular matrix proteins and old-world arenaviruses (Figure 2E) [75]. This “functional decoration” is part of a transmembrane link of the dystrophin-associated glycoprotein complex (DGC) between the extracellular matrix such as laminins, intracellular dystrophin, and the cytoskeleton, for providing resistance to sheared stresses during muscle activity. A bispecific antibody fusion could be generated, serving as a molecular linker to ameliorate sarcolemmal fragility for improving muscle function [76]. A host of enzymes such as the bifunctional glycosyltransferase LARGE are required for synthesizing α-DG mannosylation [16,77]. The local injection of recombinant α-dystroglycan (α-DG) produced by HEK293 co-transfected with LARGE rescues muscle activity in *α-DG* knockout or *Large^myd^*-mutant mice [78]. When the matriglycan-glycosylated α-DG is injected systemically, very little change is noted in muscle tissues, presumably due to the rapid clearance by the MR or ASGPR [76]. This result indicates that O-mannosylation of α-DG does interact with the extracellular matrix for therapeutic remedy but has issues of bioavailability in circulation. Further protein engineering work such as chemically conjugated matriglycan or cell-line engineering [75] is required for harnessing this modification for therapeutic applications.

## 6. Glycoengineering as a Continued Theme for Biotherapeutics Applications

Recent breakthroughs in gene editing have revolutionized glycoengineering in mammalian cells and led to improved designs of therapeutic proteins [1,6,12]. The consistent production of safer and potentially more efficacious biotherapeutics have been the primary goals for these efforts. Lysosomal glucocerebrosidase (GBA) is one of the earliest glycoengineered examples produced with high-mannose N-linked glycans for macrophages affected by GBA storage defect [79]. Broader applications of therapeutic N-glycan engineering have been the desire to optimize N-glycan α2-3-linked sialylation (Figure 1A), as N-glycan decoration with this moiety has been demonstrated to allow molecules to evade clearance by ASGPRs [80,81]. Recently, recombinant α-galactosidase used for the treatment of the lysosomal-disorder Fabry disease was produced with N-glycans having α-2,3-linked sialic acid, which has improved circulation and biodistribution with efficacy in a mouse model [82]. Through engineering additional N-linked consensus sites such as hyperglycosylated erythropoietin [83], a recombinant ENPP1-Fc for enzyme-replacement therapy showed the improved pharmacodynamics and in vivo activity of ENPP1-Fc [84]. α2-6-linked N-glycan sialylation has also been a focus of glycoengineering efforts, particularly with antibodies. Intravenous immunoglobulin (IVIG) is composed of polyclonal IgG harvested from healthy donors and is used in the treatment of autoimmune and inflammatory diseases [85]. Previous studies have demonstrated that the Fc portion of the Ig is sufficient for this anti-inflammatory activity and that this property is due to α2-6-linked sialylation on the N-glycan [86], triggering the conformational change of Fc for enabling the interaction with type II FcγRs [87]. The administration of soluble forms of glycosyltransfersases has also shown the potential as a novel strategy to treat autoimmune diseases. Recombinant soluble galactosyltransferase and α2-6-linked sialyltransferase enzymes were demonstrated to have a similar effect to IVIG on autoimmune inflammation, presumably via the in vivo glycoengineering of endogenous IgG [88]. While MGAT3 inhibits complex N-glycan branching, the overexpression of MGAT4 and MGAT5, which add β1,4- and β1,6-linked GlcNAc to the α3- and α6-arms of the N-glycan, increases the glycan antennary structure and potentially the number of sites available for sialylation [89]. Recently, the sialylation of IgE has been proposed as a determinant of allergic pathogenicity, and the treatment with neuraminidase enzyme or administering asialylated IgE might represent an interesting therapeutic strategy for allergic disease [90]. Cetuximab was shown to be recognized by IgE antibodies targeting Gal-α-1,3-Gal (αGal), demonstrating the clinical effects of immunogenic glycans and the need for glycoengineering [91]. Conversely, antibody sialidase fusions and conjugates (Figure 2D) can de-sialylate cancer cells and enhance immune responses in vivo [22,23,92].

Removing immunogenic glycans widely expressed in non-primate mammalian cells, such as αGal epitope and N-glycolyneuraminic acid, has long been a glycoengineering approach [1,10,93]. To simplify functional analysis and assignments, glycoengineering has revealed isoenzyme functions in specific steps of glycosylation pathways, including the knockout study demonstrating that ST3GAL4, not ST3GAL3/6, is the major contributor in forming the sialyl-LewisX (SLx) for lymphocyte trafficking and extravasation [94]. It also helps define virus infection requirements such as the involvement of α2-3 and α2-6 sialic acids for enterovirus D68 recognition [95,96], as well as α2-fucosyltransferase (FUT2)-modified blood group H glycan epitope for norovirus [97]. The enhancement of antibody-dependent cellular cytotoxicity via the production of afucosylated IgGs is the most widely used application for the glycan engineering of therapeutics and has been extensively reviewed [6,98,99,100,101,102]. This has been accomplished via several different mechanisms, the most well studied one being the direct knockout of the fucosyltransferase responsible for the addition of the sugar, FUT8 [103]. Alternatively, several other indirect approaches have been successfully implemented. One method is the alteration of genes coding for glycosyltransferases, of which the activity impacts that of FUT8, such as knockout of the N-acetylglucosaminyltransferase GnTI [104], or overexpression of GnTIII and variant forms thereof, which adds bisecting GlcNAc to N-glycans [105]. The targeting of the GDP-fucose donor substrate synthesis pathway has also proven successful, via the overexpression of prokaryotic enzymes, which can divert key intermediates in the GDP-fucose biosynthetic pathway into non-functional products [106,107]. In addition, the knockout of the resident Golgi GDP-fucose transporter Slc35c1 leads to the production of afucosylated recombinant proteins [108]. A number of chemical inhibitors targeting these proteins have been designed with fucose analogs such as fluoro- and thio-fucose [109,110,111,112,113,114,115]. The fluorinated fucose analogs are taken up by cells and converted via the salvage pathway to the corresponding donor substrates that can compete with the actual enzymatic substrate. The intracellular accumulation of these analogue sugars inhibit the de novo synthesis by acting as a feedback inhibitor [112,114,115]. The production of afucosylated reactive non-neutralizing IgG1 during Dengue virus infection triggers platelet reduction and is a significant risk factor for thrombocytopenia [116]. Besides modulating the interaction between IgG1 and the FcγRIIIa receptor, FUT8-mediated core fucosylation also regulates the EGF-EGFR binding [117] and T-cell receptor activation [118]. The level of core fucosylation and galactosylation in IgG can be further fine-tuned by an inducible expression strategy [119]. In addition, the terminal galactosylation of IgG plays an important role in modulating complement-dependent cytotoxicity (CDC) activity [120,121,122,123]. It has been recently demonstrated that galactosylation promotes the hexamerization of IgG1, which consequently enhances C1q binding and CDC activity [120,122].

## 7. Conclusions and Perspectives

In summary, major advances in single-cell transcriptomes and proteomes, precise glycan analytical tools, in silico modeling, vast pathway databases, and mature nuclease-based gene editing have provided an unprecedented opportunity to study the global functions of human glycome. Efficient genetic approaches such as glycoCRISPR (gene targeting for human glycosyltransferase genes [124]) and cell-based libraries of displayed glycome (GAGOme [125] and GlycoDisplay [126]), as well as chemical and metabolic glycoengineering [127], have offered simple and direct ways to explore and exploit glycosylation. Modularization strategies for the de novo interpretation of glycan structures such as StrucGP [128] should facilitate in-depth structural and functional studies on glycoproteins. These new understandings of glycan modifications in both cellular and biological systems should produce new insights into designing safer and more efficacious biotherapeutics.
